# *Salmonella enterica* serovar Ohio septic arthritis and bone abscess in an immunocompetent patient: a case report

**DOI:** 10.1186/1752-1947-6-204

**Published:** 2012-07-17

**Authors:** Hideaki Kato, Atsuhisa Ueda, Jun Tsukiji, Kayoko Sano, Mikiko Yamada, Yoshiaki Ishigatsubo

**Affiliations:** 1 Department of Internal Medicine and Clinical Immunology, Yokohama City University Graduate School of Medicine, 3-9, Fuku’ura, Kanazawa, Yokohama City, 236-0004, Kanagawa, Japan; 2 Clinical Laboratory, Yokohama City University Hospital, 3-9, Fuku’ura, Kanazawa, Yokohama City, 236-0004, Kanagawa, Japan; 3 Yokohama City Institute of Public Health, 1-2-17, Takigashira, Isogo, Yokohama City, 235-0012, Kanagawa, Japan

**Keywords:** *Salmonella* Ohio, Non-typhi salmonellosis, Extra-intestinal focal infection, Immunocompetent patient

## Abstract

**Introduction:**

Non-typhi *Salmonella* species cause severe extra-intestinal focal infection after occult bacteremia. Although the number of cases of non-typhi salmonellosis is increasing worldwide among patients with immunocompromising conditions such as human immunodeficiency virus infection, infection is uncommon in immunocompetent subjects. We report a case of septic arthritis and bone abscess due to a rare non-typhi *Salmonella* organism that developed after a prolonged asymptomatic period.

**Case presentation:**

A 44-year-old Japanese immunocompetent man presented with acute-onset left knee pain and swelling. He had no history of food poisoning, and his most recent travel to an endemic area was 19 years ago. *Salmonella enterica* serovar Ohio was identified from samples of bone abscess and joint tissue. Arthrotomy and necrotic tissue debridement followed by intravenous ceftriaxone was successful.

**Conclusions:**

Non-typhi *Salmonella* species only rarely cause extra-intestinal focal infections in immunocompetent patients. Our case suggests that non-typhi *Salmonella* species can cause severe focal infections many years after the occult bacteremia associated with food poisoning.

## Introduction

The non-typhi bacterium *Salmonella enterica* subspecies *enterica* serovar Ohio (*Salmonella* Ohio) is a rare human pathogen that can be acquired from livestock to cause zoonotic enterocolitis. Salmonellosis typically manifests as enterocolitis, with only rare extra-intestinal focal infections; the rates of osteomyelitis and septic arthritis due to *Salmonella* (typhi and non-typhi strains combined) are estimated to be less than 1% and 0.1% to 0.2% [1], respectively. Despite the reports of several cases of enterocolitis due to *Salmonella* Ohio [2-4], the only reported extraintestinal focal infection by this organism involved a soft tissue abscess [5].

Even though the prevalence of non-typhi salmonellosis in humans is increasing worldwide among patients immunocompromised due to conditions such as human immunodeficiency virus (HIV) infection, non-typhi salmonellosis remains rare in immunocompetent subjects. Although *Salmonella* species easily enter the bloodstream and may cause focal salmonellosis after occult bacteremia, routine antimicrobial therapy of immunocompetent patients with only intestinal symptoms is considered unnecessary [6].

## Case presentation

**Figure 1:**
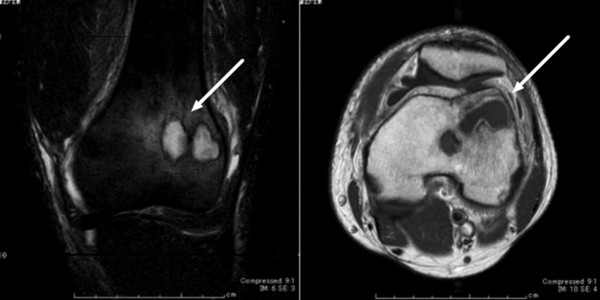
**MRI of the left knee; longitudinal scan on T2-weighted image (left panel), horizontal scan on enhanced T1-weighted image (right panel).** Polycystic bone abscess lesions were detected in the end of the left femur.

We report the case of a 44-year-old Japanese man without any noteworthy medical history (including food poisoning) except for a right tibial fracture due to a traffic accident 30 years ago. His most recent foreign travel to an endemic area (Hong Kong) was 19 years ago, and he had not noticed any gastrointestinal symptoms during that trip. He is an office worker and has no contact with livestock animals. In June 2011, he suddenly developed left knee pain that gradually worsened and therefore visited our hospital. X-rays showed no apparent bone fractures, but an MRI revealed a high-intensity area on T2-weighted images that was suggestive of a femoral bone abscess (Figure 1). Approximately 60mL of cloudy yellowish joint fluid was collected, and *Salmonella* (O:7 positive) was isolated. *In vitro* susceptibility testing by the broth microdilutionmethod using the MicroScan WalkAway 96SI system (Siemens, Germany) revealed susceptibility to ampicillin (minimal inhibitory concentration, <4μg/mL), amoxicillin–clavulanic acid (<8μg/mL), cefotaxime (<8μg/mL), imipenem (<1μg/mL), minocycline (2μg/mL), levofloxacin (<1μg/mL), and sulfamethoxazole–trimethoprim (<1μg/mL). The strain also was susceptible to nalidixic acid (30μg), according to the disc method (Becton Dickinson, Cockyesville, MD, US). Blood cultures and an HIV test were both negative. Full-body computed tomography (CT) ruled out an aortic aneurysm. In light of the diagnosis of femoral abscess with septic arthritis and the results of *in vitro* susceptibility testing, therapy with a third-generation cephalosporin (intravenous ceftriaxone 1g, once daily) was started.

On day 22 after presentation, he underwent arthrotomy and necrotic tissue resection. The synovium of his left knee joint was inflamed, and the distal end of his left femur contained multiple loculations that were filled with necrotic tissue. The necrotic tissue was debrided and the cavities filled with imipenem-loaded bone cement. Cultures of the necrotic tissue obtained during surgery grew O:7-positive *Salmonella*, as seen previously. To characterize the organism, we sent it to the Yokohama City Institute of Public Health for further analysis. The organism was classified as *Salmonella enterica* subspecies *enterica* serovar Ohio according to the serotype (6,7: b: l,w) assigned by using the Kauffmann–White scheme [7] based on agglutination with *Salmonella* O and H antigens (Denkaseiken, Tokyo, Japan). After postoperative rehabilitation, he was discharged on day 55 after presentation and received oral amoxicillin (500mg, four times daily) for six months in place of intravenous ceftriaxone.

## Discussion

Cases of non-typhi salmonellosis in immunocompromised patients are increasing worldwide. Most of these cases are gastrointestinal and systemic infections, and additional complications remain rare. One of the few reports from countries in southeastern Asia suggested age older than 60 years and younger than 6 years as independent risk factors for non-typhi salmonellosis [8]; another study from the same geographic region named age older than 50 years as a risk factor for the infectious vasculitis caused by non-typhi *Salmonella*[9]. Although food poisoning with *Salmonella* Ohio has occurred in Europe and Mexico [2-4], the organism is more prevalent in southeastern Asia, but there have been no previous clinical reports of *Salmonella* Ohio infection in Japan. One report [10] suggested that food poisoning due to non-typhi *Salmonella* has a very low incidence in Japan. In that report, the incidences of *Salmonella* gastroenteritis in the ‘Asia Pacific, High Income’ and ‘Asia, East’ regions were estimated to be 32 and 3600 per 100,000 person-years, respectively [10]. This study assigned Japan to the ‘Asia Pacific, High Income’ region. Another report suggests that non-typhi salmonellosis (especially serotype D) was still prevalent in Hong Kong during 1982 to 1993 [11]. Our patient had no history of food poisoning, and his most recent travel to another endemic country occurred 19 years before presentation. Despite the lack of microbiologic confirmation of prior infection with *Salmonella* Ohio, oral infection at some point during his residence in Japan cannot be ruled out. In-depth discussion regarding his past medical history failed to reveal any probable source of the infection. We surmise that our patient had been infected asymptomatically at some point during the preceding 19 years and that the current joint and bone infection developed long after the primary incident. The reason for, and the duration of, the asymptomatic period are currently unknown and warrant further investigation.

*Salmonella* species enter the bloodstream readily, and blood cultures should be considered whenever *Salmonella* infections are suspected or diagnosed. Routine antibiotic therapy of immunocompetent patients with only gastrointestinal symptoms of salmonellosis currently is not considered to be necessary [6]. However, perhaps this therapy should be provided, given the possibility of late serious sequellae, as in our patient. A report from Taiwan showed that the non-typhi *Salmonella* serovar Choleraesuis has low susceptibility to quinolones; therefore, empiric treatment with a third-generation cephalosporin should be considered [9]. In our patient, identifying the bacterial strain contributed to choosing the appropriate treatment for his bone infection.

## Conclusions

We presented a case of septic arthritis and bone abscess due to a rare pathogen, *Salmonella enterica* serovar Ohio, in a 44-year-old immunocompetent man with no recent history of travel abroad or food poisoning. Although routine antibiotic therapy of immunocompetent patients with gastrointestinal salmonellosis only is thought currently to be unnecessary, severe extra-intestinal focal infections can occur after a prolonged (for example, decades) asymptomatic period.

## Consent

Written informed consent was obtained from the patient for publication of this case report and any accompanying images. A copy of the written consent is available for review by the Editor-in-Chief of this journal.

## Competing interests

The authors declare that they have no competing interests.

## Authors’ contributions

HK and JT monitored our patient during hospitalization and analyzed data from the literature. KS and MY isolated and identified the organism. HK was the major contributor in writing the manuscript. AU and YI reviewed the manuscript. All authors have read and approved the final manuscript.

## References

[B1] Pegues DA Miller S Mandell GE, Douglas RG, Bennett JE Salmonella species, including typhoid fever Principles and Practice of Infectious Diseases 2010 Seventh Churchill Livingstone Inc, New York 2887 2903

[B2] Bertrand S Dierick K Heylen K De Baere T Pochet B Robesyn E Lokietek S Van Meervenne E Imberechts H De Zutter L Collard JM Lessons learned from the management of a national outbreak of Salmonella Ohio linked to pork meat processing and distribution J Food Prot 2010 73 529 534 20202340 10.4315/0362-028x-73.3.529

[B3] Paniagua GL Monroy E García-González O Alonso J Negrete E Vaca S Two or more enteropathogens are associated with diarrhoea in Mexican children Ann Clin Microbiol Antimicrob 2007 6 17 10.1186/1476-0711-6-17 18162140 PMC2246149

[B4] Soto SM Martinez N Guerra B Gonzalez-Hevia MA Mendoza MC Usefulness of genetic typing methods to trace epidemiologically Salmonella serotype Ohio Epidemiol Infect 2000 125 481 489 10.1017/S0950268800004921 11218198 PMC2869631

[B5] Macfarlane DE Multiresistant Salmonella Ohio infections at the University Hospital of the West Indies J Trop Med Hyg 1986 89 67 70 3773019

[B6] Sirinavin S Garner P Antibiotics for treating salmonella gut infections Cochrane Database Syst Rev 2000 2 CD001167 10796610 10.1002/14651858.CD001167

[B7] Grimont PAD Weill F Antigenic Formulae of the Salmonella Serovars 2009 9 Institut Pasteur, Paris

[B8] Lee WS Hafeez A Hassan H Raja NS Puthucheary SD Focal non-typhoidal Salmonella infections from a single center in Malaysia Southeast Asian J Trop Med Public Health 2005 36 678 682 16124437

[B9] Chen PL Wu CJ Chang CM Lee HC Lee NY Shih HI Lee CC Ko NY Wang LR Ko WC Extraintestinal focal infections in adults with Salmonella enterica serotype Choleraesuis bacteremia J Microbiol Immunol Infect 2007 40 240 247 17639165

[B10] Majowicz SE Musto J Scallan E Angulo FJ Kirk M O’Brien SJ Jones TF Frazil A Hoekstra RM The global burden of nontyphoidal Salmonella gastroenteritis Clin Infect Dis 2010 50 882 889 10.1086/650733 20158401

[B11] Ling JM Koo IC Kam KM Cheng AF Antimicrobial susceptibilities and molecular epidemiology of salmonella enterica serotype enteritidis strains isolated in Hong Kong from 1986 to 1996 J Clin Microbiol 1998 36 1693 1699 9620402 10.1128/jcm.36.6.1693-1699.1998 PMC104902

